# A cross-over, randomised feasibility study of digitally-printed versus hand-painted artificial eyes in adults: PERSONAL-EYE-S

**DOI:** 10.1038/s41433-024-03273-0

**Published:** 2024-08-02

**Authors:** Amie Woodward, Elizabeth Coleman, Sarah Ronaldson, Tim Zoltie, Paul Bartlett, Laura Wilson, Tom Archer, Jessica Kawalek, Florien Boele, Bernard Chang, George Kalantzis, Mike Theaker, Nabil El-Hindy, Emma Walshaw, Taras Gout, Judith Watson

**Affiliations:** 1 York Trials Unit, Department of Health Sciences, University of York, York, UK; 2 School of Dentistry, Faculty of Medicine and Health, University of Leeds, Leeds, UK; 3 Maxillofacial Laboratory, Leeds Dental Institute, Leeds Teaching Hospitals NHS Trust, Leeds, UK; 4 Leeds Artificial Eye Service, Leeds Dental Institute, Leeds Teaching Hospitals NHS Trust, Leeds, UK; 5 Leeds Institute of Medical Research at St James’s, St James’s University Hospital, University of Leeds, Leeds, UK; 6 Leeds Institute of Health Sciences, Faculty of Medicine and Health, University of Leeds, Leeds, UK; 7 Department of Ophthalmology, Leeds Teaching Hospitals NHS Trust, Leeds, UK; 8 Patient and Public Involvement representative, Leeds, UK

**Keywords:** Outcomes research, Education

## Abstract

**Background/objectives:**

Over 60,000 patients in the United Kingdom are estimated to have artificial eyes. Manufacturing and hand-painting of artificial eyes have not changed significantly since 1948. Delays and colour-matching issues may severely impact a patient’s rehabilitation pathway. Technology advances mean alternatives are now possible. This cross-over, randomised feasibility trial aimed to determine the feasibility of conducting a full-scale trial of the effectiveness and cost-effectiveness of digitally-printed artificial eyes compared to hand-painted.

**Subjects/methods:**

Patients aged ≥18 years who were longstanding artificial eye users requiring a replacement were randomised to receive either a hand-painted or digitally-printed eye first followed by the other type of eye. Participants were asked to approach a close contact (CC) willing to participate alongside them. A subset of participants, their CCs, and staff were interviewed about their opinions on trial procedures, artificial eyes, delivery times and satisfaction.

**Results:**

Thirty-five participants were randomised and 10 CCs consented. Participant retention at final follow-up was 85.7%. Outcome data completion rates ranged from 91–100%. EQ-5D-5L completion ranged from 83–97%. Resource-use completion ranged from 0–94% with total costs at £347 for hand-painted and £404 for digitally-printed eye. There were two adverse events. Twelve participants, five CCs, and five staff were interviewed. There were positive and negative features of both types of eyes. We identified that social and psychological wellbeing is affected, often for many years after eye removal. Participation in the feasibility study was well accepted.

**Conclusions:**

The feasibility study outcomes indicate that a full trial is achievable.

**Trial registration number:**

ISRCTN85921622.

## Introduction

In the United Kingdom (UK), around 1,400 people have surgery to remove their eye each year and an estimated 60,000 patients have an artificial eye [1]. The National Artificial Eye Service (NAES) and approximately 30 local artificial eye services provide replacement artificial eyes every 2–6 years for patients, totalling around 11,500 artificial eyes per year. This demand can cause pressure on services and result in delays to the provision of artificial eyes.

Many patients suffer from anxiety and depression associated with surgeries to remove their eye and perceived post-operative disfigurement until a realistic artificial eye is achieved [2–4]. This demonstrates the important role of a well-fitted, life-like artificial eye in the rehabilitation journey to enhance patients’ health-related quality of life (HRQoL).

The predominant method used worldwide, and by NAES since 1948, is hand-painted artificial eyes. The eye is manufactured at a centralised site in 6–10 weeks and colour-matched using non-standardised samples. However, achieving a good colour match is difficult and often requires multiple revisions to achieve a realistic artificial eye that is acceptable to patients. Patient and Public Involvement (PPI) representatives have reported this process can take up to one year. And as a result, causes them distress and delays their return to a normal home, social and work life.

In response, Leeds Artificial Eye Service (LAES) (Leeds, UK) identified an unmet need to improve patient rehabilitation by manufacturing realistic artificial eyes in a shorter time frame. This was proposed by utilizing digital colour-matching and printing [5]. Preliminary work demonstrated that patients could receive a more life-like match, often within 2 weeks, and require fewer clinic visits. Digital photography in the manufacture of artificial eyes has been described previously, although this was restricted to just the iris and not the entirety of the visible eye [6].

Further research is needed to determine whether digitally-printed eyes result in improvements in patients’ HRQoL and satisfaction. Since a large-scale randomised controlled trial (RCT) would be needed to provide answers on those aspects and overall costs, this study aimed to examine the feasibility of conducting one.

## Aims and objectives

The primary aim of this study was to determine the feasibility of conducting an RCT of the effectiveness and cost-effectiveness of digitally-printed artificial eyes compared to hand-painted eyes.

The specific objectives were to:

- Determine the number of patients meeting the eligibility criteria;
- Determine the patient recruitment rate including barriers to patient enrolment, proportion providing consent, reasons for non-consent, proportion withdrawing and reasons why (where possible);
- Identify attrition rates, data fidelity and missing data;
- Identify a primary outcome measure(s) for a future trial (if feasibility established);
- Test study procedures and data collection tools and management;
- Establish scalability of the current service.

A full RCT is deemed feasible if:

- Patient recruitment and retention rates indicate recruitment for a full-scale RCT is plausible;
- Outcome measures and fidelity evaluation data are successfully collected. Measures with over 10% missing data may be modified/replaced prior to the main trial;
- Qualitative data confirms willingness of patients to be recruited, randomised and find research processes acceptable; healthcare professionals’ opinions on the different artificial eyes, views on delivery times and patient satisfaction prove acceptable.

## Subjects and methods

### Study design

A cross-over, randomised controlled, open feasibility study conducted in one National Health Service (NHS) site. The methods are summarised below and described in full elsewhere [7]. This study is reported according to the relevant CONSORT guidelines [8, 9]. Being a feasibility study, the primary outcome measure does not inform a sample size calculation, but one objective is to estimate the within-subject standard deviation for each outcome measure, in order to inform the sample size calculation for a future study. Literature on pilot and feasibility trials recommends a sample size of between 24 and 70 to inform reliable estimation of standard deviations [10, 11]. Thus, we aimed to recruit 35 participants, assuming a 15% attrition rate, allowing for 30 participants in the final analysis.

### Patients and setting

Prospective participants were identified in LAES clinic, via database screening or notification placed on the Royal National Institute of Blind People (RNIB) and Blind Veterans UK websites. Patients were provided with an invitation pack and asked to return a Consent to Contact form to York Trials Unit (YTU) if interested in the trial. Upon receipt of this form, YTU contacted patients to discuss any queries and arrange an eligibility assessment with LAES if appropriate.

Interested patients were also asked to approach a close contact (CC) (e.g. friend, family member), who might be willing to participate alongside them. CCs were provided with their own information pack and were required to provide consent to participate. Patients could take part without a CC, but not vice versa.

### Eligibility criteria

Longstanding artificial eye users (AEUs) (≥12 months post-operation), aged ≥ 18 years old, who required a replacement artificial eye and were able to complete the English language outcome measures (independently or with assistance) were eligible to participate. Those with ongoing clinical concerns regarding their artificial eye use (e.g. poor socket healing, extrusion, dehiscence), bilateral artificial eyes, pregnant or persons currently shielding (to avoid unnecessary clinic visits due to the ongoing pandemic at the time) or participating in another artificial eye study, were excluded.

LAES developed the novel artificial eye manufacture method and service provision technique over several years. This involved a number of service evaluations to help refine it over time. As an open, non-masked study we accepted all patients to our study including those with previous digitally-printed and hand-painted artificial eyes.

### Clinics and interventions

Potential participants attended an initial clinic appointment (Clinic 1) where eligibility was confirmed, written informed consent obtained and baseline data collected, including demographics, past medical and family history relating to eye health, ocular and artificial eye history, past and current mental health issues, medications and allergies. A baseline questionnaire pack containing EQ-5D-5L, Short-Form 36 (SF-36), Vision Quality of Life Index (VisQol), Connor–Davidson Resilience Scale (CD-RISC10), Derriford Appearance Scale (DAS-24) short form, as well as health resource use questions were completed. Further details are available in the published protocol [7].

Patients were then individually randomised on a 1:1 basis using block randomisation (no stratification) via a secure internet-based service hosted by the UKCRC-accredited YTU to a) receive a digitally-printed artificial eye first (intervention), followed by a hand-painted artificial eye (control); or b) receive a hand-painted artificial eye first, followed by a digitally-printed artificial eye. The first allocated eye was fitted at the second appointment (Clinic 2).

After wearing the first eye for ~2 weeks, participants attended Clinic 3 (i.e. a 3rd appointment) to complete the same questionnaires as at baseline, with the addition of satisfaction questions. Clinical data was also collected on socket health and adverse events. During Clinic 4, the second eye was fitted and then following wearing of that second eye, data collection was repeated at Clinic 5. At these same time points (Clinics 3 and 5), participating CCs were asked to complete a satisfaction questionnaire. At Clinic 5, participants and CCs were asked to compare photographs (taken by Trust medical and dental imaging team specialist photographers) of both eyes worn by the participant and state a preference. Clinic staff compared photographs for each randomised participant at the end of the trial, stating their preference.

### Analyses

The number of patients screened, eligible, consented and randomised were summarised. Continuous data were reported descriptively (mean, standard deviation, median, minimum and maximum) and categorical data by counts and percentages. All measures were scored and completion rates detailed.

The feasibility of undertaking an economic evaluation from an NHS perspective was explored. We aimed to develop an appropriate economic evaluation framework, identify relevant health economic data and consider the feasibility of data collection methods to inform a future full trial. This work explored individual patient-level data regarding the EQ-5D-5L, resource use and costs of the two eye services. Full details of the health economics methods and analysis will be available elsewhere (*manuscript under consideration*).

### Qualitative data collection and analyses

After both eyes had been trialled, we aimed to conduct semi-structured interviews (telephone, video-call or in person), using topic guides, with approximately 15 participants and their CCs (up to 15) to gain an in-depth understanding of the acceptability of the trial procedures (including recruitment, consent, randomisation, clinic visits and data collection) and how each artificial eye impacted on patients’ quality of life and wellbeing. Participants were purposively selected from those who agreed to be approached for interview, to cover the perspectives of men/women, of different ages, who suffered eye loss for a variety of reasons and with a range of time since eye loss [12]. In addition, staff involved in the research were invited to be interviewed during the later parts of the study focussing on their opinions on the different artificial eyes, including views on delivery times and patient satisfaction.

All interviews were digitally recorded with permission, transcribed and analysed thematically.

## Results

### Recruitment

**Fig. 1:**
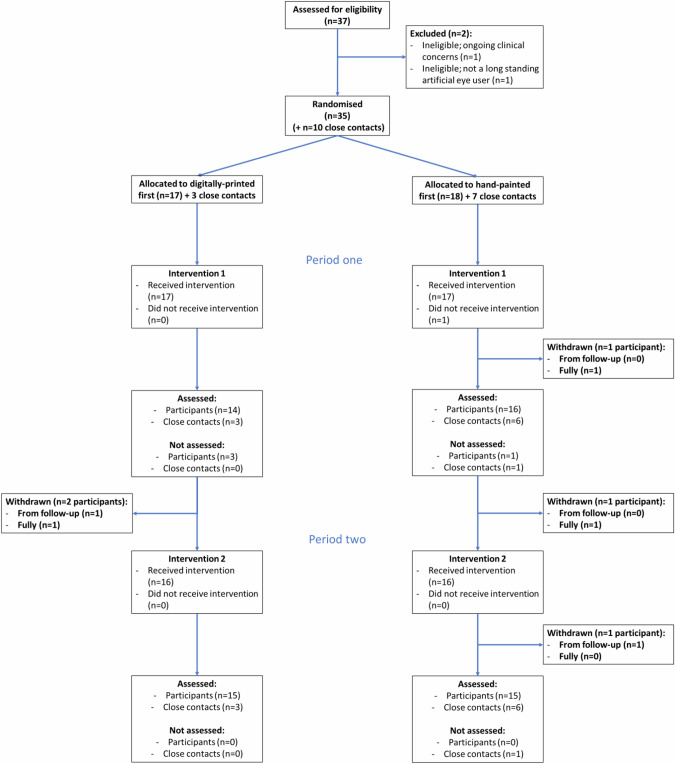
CONSORT flow diagram. The diagram shows the number of participants assessed for eligibility, reasons for exclusion and number randomised. Numbers who received treatments as allocated, were lost to follow up and numbers included in the analysis are also shown.

Between October 2021 and June 2022, 50 consents to contact (CtoCs) were received from patients interested in participating in the trial, including three from people directly contacting the research team. Of these, two people declined participation after speaking with the trial team, one person was uncontactable. From these, 37 (78.7% of CtoCs) were formally screened and 35 (94.6% of screened) were eligible and consented to participate (100%). Of the ineligible participants: one had ongoing clinical issues and the other was not a longstanding artificial eye user. We recruited 3.9 participants per month. Ten CCs were recruited (28.6% of participants). Figure 1 shows the CONSORT flow diagram.

### Participants

The mean age was 52.8 years (SD 17.2; range 21–89), 21 (60%) were male and the majority (82.9%) were of a white British ethnicity. On average it had been 22.6 years since their eye removal (range 1.3–66.7 years). The majority (*n* = 31, 88.6%) were currently using a hand-painted prosthesis. Participants reported a range of mental health conditions linked to their eye loss including depression (*n* = 4), post-traumatic stress disorder (*n* = 5) and anxiety (*n* = 5). Table 1 shows a summary of baseline characteristics of the randomised participants.

**Table 1:** Baseline characteristics of the participants, as randomized.

	Digitally-printed first (*n* = 17)	Hand-painted first (*n* = 18)	Overall (*n* = 35)
Age, years	*N* = 17	*N* = 18	*N* = 35
Mean (SD)	47.7 (18.0)	57.7 (15.4)	52.8 (17.2)
Median (min., max.)	42 (21, 79)	60 (33, 89)	51 (21, 89)
Gender, *n* (%)			
Male	12 (70.6)	9 (50.0)	21 (60.0)
Female	5 (29.4)	9 (50.0)	14 (40.0)
Prefer not to say	0 (0.0)	0 (0.0)	0 (0.0)
Other	0 (0.0)	0 (0.0)	0 (0.0)
Ethnic background, *n*(%)^a^			
White British	14 (82.4)	15 (83.3)	29 (82.9)
Asian Pakistani	0 (0.0)	1 (5.6)	1 (2.9)
White & Black African	1 (5.9)	0 (0.0)	1 (2.9)
White & Asian	0 (0.0)	1 (5.6)	1 (2.9)
Other	1 (5.9)	1 (5.6)	2 (5.7)
No data	1 (5.9)	0 (0.0)	1 (2.9)
Marital Status, *n*(%)			
Living alone, never married	3 (17.7)	1 (5.6)	4 (11.4)
Living with partner	3 (17.7)	7 (38.9)	10 (28.6)
Married/civil partnership	7 (41.2)	7 (38.9)	14 (40.0)
Separated	0 (0.0)	1 (5.6)	1 (2.9)
Divorced	3 (17.7)	0 (0.0)	3 (8.6)
Widowed	0 (0.0)	2 (11.1)	2 (5.7)
No data	1 (5.9)	0 (0.0)	1 (2.9)
Main activity, *n*(%)			
Full-time employment	6 (35.3)	3 (16.7)	9 (25.7)
Part-time employment	1 (5.9)	0 (0.0)	1 (2.9)
Self-employed	3 (17.7)	3 (16.7)	6 (17.1)
Unable to work—ill health	1 (5.9)	3 (16.7)	4 (11.4)
Unemployed	1 (5.9)	0 (0.0)	1 (2.9)
Retired	3 (17.7)	8 (44.4)	11 (31.4)
Student	0 (0.0)	0 (0.0)	0 (0.0)
Housework	1 (5.9)	0 (0.0)	1 (2.9)
Other	0 (0.0)	0 (0.0)	0 (0.0)
No data	1 (5.9)	1 (5.6)	2 (5.7)
Living arrangements, *n*(%)			
Owner occupied—outright	2 (11.8)	7 (38.9)	9 (25.7)
Owner occupied—mortgage	8 (47.1)	2 (11.1)	10 (28.6)
Rented (Council/housing association)	4 (23.5)	5 (27.8)	9 (25.7)
Privately rented	2 (11.8)	3 (16.7)	5 (14.3)
Temporary accommodation	0 (0.0)	0 (0.0)	0 (0.0)
Residential/Nursing home	0 (0.0)	0 (0.0)	0 (0.0)
No data	1 (5.9)	1 (5.9)	2 (5.7)
Time since eye removal, years	*N* = 17	*N* = 17	*N* = 34
Mean (SD)	16.8 (13.9)	28.4 (22.9)	22.6 (19.6)
Median (min., max.)	12.5 (1.3, 43.4)	20.0 (3.6, 66.7)	18.3 (1.3, 66.7)
Eye removal reason, *n*(%)			
Medical reason	11 (64.7)	8 (44.4)	19 (54.3)
Trauma/accident	6 (35.3)	10 (55.6)	16 (45.7)
Current prosthesis, *n*(%)			
Digitally-printed	2 (11.8)	2 (11.1)	4 (11.4)
Hand-painted	15 (88.2)	16 (88.9)	31 (88.6)
Family medical history, *n*(%)^b^			
High blood pressure	2 (11.8)	7 (38.9)	9 (25.7)
Macular degeneration	2 (11.8)	2 (11.1)	4 (11.4)
Diabetes	5 (29.4)	6 (33.3)	11 (31.4)
Cancers affecting the eye	0 (0.0)	0 (0.0)	0 (0.0)
Glaucoma	5 (29.4)	3 (16.7)	8 (22.9)
Retinal detachment	2 (11.8)	1 (5.6)	3 (8.6)
Other: cataracts	0 (0.0)	1 (5.6)	1 (2.9)

*min.* minimum, *max.* maximum, *SD* standard deviation.

^a^Empty responses omitted.

^b^Multiple options could be selected.

There were five withdrawals within the trial—two from follow-up and three full withdrawals (14.3% of 35). Full withdrawals occurred due to an adverse event, long-term illness and mental health-related issues. Follow-up withdrawals were both due to participants being repeatably uncontactable.

### Follow-up and clinic attendance

At their first follow-up clinic appointment, 34 participants were still in the trial and 30 completed the questionnaire (88.2% of those expected, 85.7% of randomised) (see 'Adverse Events' for details on the participant withdrawn prior to follow-up one). Non-completion was due to participants either being uncontactable for arranging clinical visits or due to ill health—see Withdrawals. At follow-up two, 30 participants were still participating and all completed the follow-up (100% of those expected, 85.7% of randomised). Of the CCs, 9/10 completed the follow-ups at each timepoint—with one CC completing neither follow-up. An attrition rate of ~15% was seen in this trial.

Attendance at the clinics was above 90% at each timepoint (C1 100%, C2 100%, C3 91%, C4 97% and C5 100% - of those still in the trial). For efficiency, it was decided that Clinic 3 and Clinic 4 could be combined in some instances, which may explain the lower rates at these time points. Full details can be seen in Table A in the Supplementary material.

### Outcome measures

The baseline scores and combined follow-up (i.e. the results after wearing the hand-painted eye are collated, regardless of when it was worn) scores for each outcome can be seen in Table 2, details on the outcomes at each timepoint are given in Table B in Supplementary material. The raw scores of the outcomes were similar at baseline and at follow-up for the two trial arms.

**Table 2:** Raw scores of the baseline and combined follow-up scores for each outcome measured, by arm and overall.

	Digitally-printed first (*n* = 17)	Hand-painted first (*n* = 18)	Overall (*n* = 35)
SF-36 Physical (range 0–100; higher scores are better)			
Baseline			
N	16	16	32
Mean (SD)	53.3 (6.6)	49.4 (9.8)	51.3 (8.4)
Median (min, max)	54.8 (38.6, 61.7)	52.9 (32.3, 60.0)	54.8 (32.3, 61.7)
Follow-ups: Combined			
	Digitally-printed	Hand-painted first	Overall
N	27	29	–
Mean (SD)	53.3 (7.4)	51.9 (8.6)	– (–)
Median (min, max)	55.7 (34.9, 61.5)	53.8 (27.5, 61.9)	– (–, –)
SF-36 Mental (range 0–100; higher scores are better)			
Baseline			
N	16	16	32
Mean (SD)	43.7 (17.3)	46.2 (10.2)	44.9 (14.0)
Median (min, max)	50.3 (11.0, 63.5)	48.6 (20.3, 57.7)	48.8 (11.0, 63.5)
Follow-ups: Combined			
	Digitally-printed	Hand-painted	Overall
N	27	29	–
Mean (SD)	46.3 (11.3)	48.3 (10.2)	– (–)
Median (min, max)	47.1 (21.7, 61.0)	53.1 (29.0, 63.5)	– (–, –)
VisQol (range 0–1; higher scores are worse)			
Baseline			
N	17	18	35
Mean (SD)	0.81 (0.20)	0.75 (0.31)	0.78 (0.26)
Median (min, max)	0.91 (0.38, 0.99)	0.91 (0.04, 0.99)	0.91 (0.04, 1.00)
Follow-ups: Combined			
N	29	30	–
Mean (SD)	0.85 (0.20)	0.86 (0.15)	– (–)
Median (min, max)	0.91 (0.15, 1.00)	0.91 (0.43, 1.00)	– (–, –)
CD-RISC-10 (range 0–40; higher scores are better)			
Baseline			
N	17	18	35
Mean (SD)	26.8 (8.7)	26.8 (9.3)	26.8 (8.9)
Median (min, max)	28.0 (13.0, 38.0)	27.0 (3.0, 40.0)	27.0 (3.0, 40.0)
Follow-ups: Combined			
N	27	30	–
Mean (SD)	28.7 (8.3)	27.8 (9.1)	– (–)
Median (min, max)	28.0 (13.0, 40.0)	28.0 (8.0, 40.0)	– (–, –)
DAS-24 (range 11–96, higher scores are worse)			
Baseline			
N	17	15	32
Mean (SD)	38.6 (18.6)	40.3 (22.1)	39.4 (19.9)
Median (min, max)	33 (12, 76)	33 (17, 102)	33 (12, 102)
Follow-ups: Combined			
N	29	31	–
Mean (SD)	38.7 (14.1)	37.9 (13.2)	– (–)
Median (min, max)	36 (15, 74)	35 (16, 72)	– (–, –)

*min.* minimum, *max.* maximum, *SD* standard deviation.

### Completion rates

The completion rates for each outcome varied between 91 and 100%—of the questionnaires returned. At baseline the SF-36 and DAS-24 were completed to a scorable level by 91% of responders (32/35), and the VisQol and CD-RISC-10 were completed by 100% (35/35). Similarly, at follow-up one the SF-36 had 93% completion (28/30), the VisQol and CD-RISC-10 had 90% completion (27/30) and DAS-24 had 100% completion (30/30). At follow-up 2, the SF-36 had 93% completion, and the other three outcome measures were all 100% completed (30/30). There were instances where only a few questions were missed, impacting scoring, or some instances where the whole measure was not completed, even though the questionnaire pack had been returned.

### Satisfaction

Similar levels of satisfaction were reported for both artificial eyes when asked about satisfaction in general and in relation to waiting time. A higher proportion were unhappy with the appearance of the digitally-printed (17.1%) than the hand-painted (5.7%); and more were satisfied with the fit of the hand-painted (45.7%) than the digitally-printed (31.4%).

When asked about the eye in general, CCs most commonly reported that they were ‘neither satisfied or dissatisfied’ for the hand-painted eye, and most commonly reported ‘entirely satisfied’ for the digitally-printed eye (*n* = 4/9, 44.4% for both). More CCs reported that they were ‘entirely satisfied’ with the appearance of the digital-eye (4/9), compared to the hand-painted eye (2/9). Opinions of the CCs on wait-time for the eye were similar, 8/9 CCs reporting ‘entirely satisfied’ for both eyes.

This suggests that the CCs are more satisfied with the appearance of, and in general, with the digitally-printed eye, than the hand-painted eye—but these results should be interpreted with caution, as the sample of CCs is small (*n* = 9). Table 3 summaries participant and close contact satisfaction levels.

**Table 3:** Participant and close contact satisfaction, summarised for each intervention and overall.

	Digitally-printed		Hand-painted		Overall	
	Participant (*n* = 35)	Close contact (*n* = 9)	Participant (*n* = 35)	Close contact (*n* = 9)	Participant (*n* = 70)	Close contact (*n* = 18)
How satisfied are you with the/the patient’s eye in general? *n*(%)						
Not satisfied at all	3 (8.6)	2 (22.2)	3 (8.6)	2 (22.2)	6 (8.6)	4 (22.2)
Somewhat dissatisfied	5 (14.3)	0 (0.0)	1 (2.9)	0 (0.0)	6 (8.6)	0 (0.0)
Neither satisfied nor dissatisfied	7 (20.0)	2 (22.2)	8 (22.9)	4 (44.4)	15 (21.4)	6 (33.3)
Somewhat satisfied	5 (14.3)	1 (11.1)	9 (25.7)	1 (11.1)	14 (20.0)	2 (11.1)
Entirely satisfied	9 (25.7)	4 (44.4)	10 (28.6)	1 (11.1)	19 (27.1)	5 (27.8)
No data	6 (17.1)	0 (0.0)	4 (11.4)	1 (11.1)	10 (14.3)	1 (5.6)
How satisfied are you with the fit (comfort) of the eye? *n*(%)						
Not satisfied at all	3 (8.6)	–	0 (0.0)	–	3 (4.3)	–
Somewhat dissatisfied	2 (5.7)	–	2 (5.7)	–	4 (5.7)	–
Neither satisfied nor dissatisfied	2 (5.7)	–	3 (8.6)	–	5 (7.1)	–
Somewhat satisfied	11 (31.4)	–	10 (28.6)	–	21 (30.0)	–
Entirely satisfied	11 (31.4)	–	16 (45.7)	–	27 (38.6)	–
No data	6 (17.1)	–	4 (11.4)	–	10 (14.3)	–
How satisfied are you with the appearance of the/the patient’s eye? *n*(%)						
Not satisfied at all	6 (17.1)	2 (22.2)	2 (5.7)	2 (22.2)	8 (11.4)	4 (22.2)
Somewhat dissatisfied	7 (20.0)	0 (0.0)	4 (11.4)	2 (22.2)	11 (15.7)	2 (11.1)
Neither satisfied nor dissatisfied	5 (14.3)	3 (33.3)	6 (17.1)	1 (11.1)	11 (15.7)	4 (22.2)
Somewhat satisfied	4 (11.4)	0 (0.0)	12 (34.3)	2 (22.2)	16 (22.9)	2 (11.1)
Entirely satisfied	7 (20.0)	4 (44.4)	7 (20.0)	2 (22.2)	14 (20.0)	6 (33.3)
No data	6 (17.1)	0 (0.0)	4 (11.4)	0 (0.0)	10 (14.3)	0 (0.0)
How satisfied are you with the waiting time for the/the patient’s eye? *n*(%)						
Not satisfied at all	0 (0.0)	0 (0.0)	0 (0.0)	0 (0.0)	0 (0.0)	0 (0.0)
Somewhat dissatisfied	1 (2.9)	0 (0.0)	0 (0.0)	0 (0.0)	1 (1.4)	0 (0.0)
Neither satisfied nor dissatisfied	2 (5.7)	0 (0.0)	2 (5.7)	1 (11.1)	4 (5.7)	1 (5.6)
Somewhat satisfied	4 (11.4)	1 (11.1)	4 (11.4)	0 (0.0)	8 (11.4)	1 (5.6)
Entirely satisfied	22 (62.9)	8 (88.9)	24 (68.6)	8 (88.9)	46 (65.7)	16 (88.9)
No data	6 (17.1)	0 (0.0)	4 (11.4)	0 (0.0)	10 (14.3)	0 (0.0)
How satisfied are you with your/the patient’s participation in this research study? *n*(%)						
Not satisfied at all	0 (0.0)	0 (0.0)	1 (2.9)	0 (0.0)	1 (1.4)	0 (0.0)
Somewhat dissatisfied	0 (0.0)	0 (0.0)	0 (0.0)	0 (0.0)	0 (0.0)	0 (0.0)
Neither satisfied nor dissatisfied	4 (11.4)	0 (0.0)	3 (8.6)	0 (0.0)	7 (10.0)	0 (0.0)
Somewhat satisfied	6 (17.1)	3 (33.3)	5 (14.3)	0 (0.0)	11 (15.7)	3 (16.7)
Entirely satisfied	19 (54.3)	6 (66.7)	22 (62.9)	9 (100.0)	41 (58.6)	15 (83.3)
No data	6 (17.1)	0 (0.0)	4 (11.4)	0 (0.0)	10 (14.3)	0 (0.0)
How satisfied are you with your participation in this research study? *n*(%)						
Not satisfied at all	–	0 (0.0)	–	0 (0.0)	–	0 (0.0)
Somewhat dissatisfied	–	0 (0.0)	–	1 (11.1)	–	1 (5.6)
Neither satisfied nor dissatisfied	–	0 (0.0)	–	0 (0.0)	–	0 (0.0)
Somewhat satisfied	–	3 (33.3)	–	0 (0.0)	–	3 (16.7)
Entirely satisfied	–	6 (66.7)	–	8 (88.9)	–	14 (77.8)
No data	–	0 (0.0)	–	0 (0.0)	–	0 (0.0)

### Preference

This part of the trial was done with eye-band photographs, which are cropped to just show the eyes. One of the images would have the patient wearing one artificial eye (for example the digital) and the image below it would have them wearing the other eye (hand-painted). Respondents were masked and not told which image was which eye. When asked to pick which eye they preferred participants preferred the hand-painted (44%) over the digitally-printed (36%); as did CCs (75% v 12.5%). The clinic staff had a similar level of preference (37.6% preferred hand-painted, 34.5% preferred digitally-printed).

Additionally, participants and CCs were asked which eye they preferred the appearance of (unmasked). These results mirrored the masked results, with the hand-painted eye being preferred (43.3% of participants, 55.6% of CCs). Full details are presented in Table 4.

**Table 4:** Eye preference from participants, close contacts and clinic staff.

	Preferred digitally-printed	Preferred hand-painted	Liked both the same	Liked neither
Unblinded				
Participants (*n* = 30 respondents)	12 (40.0)	13 (43.3)	2 (6.7)	3 (10.0)
Close contacts (*n* = 9 respondents)	2 (22.2)	5 (55.6)	1 (11.1)	1 (11.1)
Blinded				
Participants (*n* = 25 respondents)	9 (36.0)	11 (44.0)	3 (12.0)	2 (8.0)
Close contacts (*n* = 8 respondents)	1 (12.5)	6 (75.0)	1 (12.5)	0 (0.0)
Clinic staff (*n* = 9 assessed 255 sets of eyes—from 29 participants)	88 (34.5)	96 (37.6)	34 (13.3)	37 (14.5)

Patient inclusiveness was prioritised throughout the study. This included: the RNIB printing the patient information leaflet in Braille for one patient; ensuring the clinics were fully accessible to wheelchair users with disabled parking nearby; provision of information regarding public transport to the hospital and providing financial assistance for it based on individual needs; and, working with patients who requested specific times and re-scheduling of clinics to accommodate childcare and other caregiver commitments.

### Health economics

High response rates were observed for the EQ-5D-5L across the three time points: complete EQ-5D-5L responses were provided for 34 participants (97%) at baseline, 29 (83%) at follow-up 1 and 30 (86%) at follow-up 2. Baseline utility levels were found to be similar between the groups (0.74 on average). After wearing the hand-painted eye, mean utility increased to 0.77 whereas mean utility increased to a higher level of 0.83 after the digitally-printed eye had been trialled. Response rates were lower for the resource use questions, with none fully completed at baseline or follow-up and responses ranging from 0 to 94%. The resource use items appeared to capture the health services used by this population, though some would be removed in a full trial due to low uptake. Response to the clinical record booklet, for estimation of the eye service costs, was very good overall.

Although manufacturing times were found to be 60 min shorter, on average, for digitally-printed eyes, more appointments for re-makes were required than hand-painted eyes: nine versus none, respectively. The hand-painted eye service cost £347, whilst the digitally-printed eye service cost £404, based on the time taken at the clinics attended and incorporating re-make time (2021 prices). In addition, nine booked appointments were not attended. The interval between clinics, i.e. between fitting and final evaluation of the eye, was similar: 60 days for hand-painted and 56 days for digitally-printed.

### Adverse events

There was one non-serious adverse event (NSAE) and one serious adverse event (SAE) in total. The NSAE involved an infected left eye socket after 3 days of wearing new eye which was treated with antibiotic drops. The participant suggested they had been removing the eye and passing it around family to show them. They remained in the trial and the event was deemed possibly related but expected. The participant with the SAE arrived in clinic to have their new artificial eye fitted in their right socket. However, they complained of pain and sight loss in their left eye which was found to be red and swollen. They had had previous corneal surgery, with their last check up only the previous week. The clinic sent them to A&E. Their remaining eye required removal by surgery. This was deemed unrelated to the trial but resulted in a full withdrawal, prior to the participant receiving either invention.

### Qualitative interviews

Twelve participants, five CCs and five staff members were interviewed by a research assistant experienced in qualitative research (JK). Qualitative results related to AEU and their family members’ quality of life and day-to-day functioning have been published separately [12], with results related specifically to the trial summarised below.

AEUs and CCs generally took part in the trial for altruistic purposes (improve future services) and AEUs appreciated the opportunity to test different eyes. AEUs, CCs and HCPs highlighted both strengths and weaknesses of the two types of trialled eyes. All groups were impressed by the digitally-printed eyes’ detail and accuracy of colour matching, e.g.: *'The digital one was a very deep colour; it was very impressive how clear it was and how good the colours were'* [AEU, male, aged 34]. HCPs favoured the digitally-printed eyes’ consistency of results and quick production times but highlighted issues in suitability for AEUs without full eye sockets. Some AEUs noted that if their functioning eye was sore, the digital image would reflect that. The hand-painted eyes were also thought to have a life-like level of detail (AEUs and CCs), with some AEUs preferring their appearance, although durability and comfort were sometimes questioned: '*The last painted one tended to irritate my eyeball*' [AEU, female, aged 79].

A good match generally boosted AEUs confidence and wellbeing: '*I’ve got this perfect eye now… it’s really made me feel a lot better about myself*' [AEU, male, aged 32]. Conversely, if the trial eye was found unsuitable (e.g. inaccurate colour), it could make AEUs feel uncomfortable, especially in social situations.

AEUs and CCs generally felt the trial was well-delivered: '*There’s been no waiting around*' [AEU, female, aged 51]. The continuity of care was appreciated. Suggestions for improvements for the full-scale trial were identified by all groups. HCPs preferred more workspace and fewer clinics. Both HCPs and AEUs recommended creating a new mould at the start of the trial. AEUs and CCs suggested to reduce the length of questionnaires, limiting repetition of similar items, to allow tailoring through open-ended questions and allowing online completion.

### Feasibility outcomes

We established it is feasible to conduct a larger-scale study based on our initial criteria.

The feasibility study was deemed successful as:

- patient recruitment completed and achieved an acceptably low drop-out rate (35 patients recruited, with a 15% attrition rate);
- study consent and retention rates indicate recruitment for a full-scale RCT is plausible;
- outcome measures and fidelity evaluation data are successfully collected. Measures with over 10% missing data may be modified/replaced prior to the main trial.
- Qualitative data confirms willingness of patients to be recruited, randomised and find research processes acceptable; and healthcare professionals’ opinions on the different artificial eyes, views on delivery times and patient satisfaction prove acceptable.

## Discussion

Creating a more life-like artificial eye in a shorter time period and reducing costs is an important goal. This should in turn improve our patients’ initial rehabilitation pathway, long-term quality of life and service experience. This feasibility study suggests a large-scale superiority crossover randomised controlled trial is feasible. The aim is for this research to transition into patient benefit initially locally and later for the wider NHS across the UK.

Whilst no formal comparisons have been made in this feasibility study, baseline scores were similar in both groups across the outcome measures used. Similarities were also seen for the combined follow-up mean scores for VisQoL, CD-RISC-10 and DAS-24, suggesting the participants’ abilities to cope with various aspects of their lives, levels of resilience and concerns about their appearance were not impacted by the type of artificial eye. However, there was a larger difference when comparing the mean scores of the mental component of SF-36 between the two groups with the hand-painted first group faring better.

Participants and CCs appeared to prefer the hand-painted eye over the digitally-printed eye when viewing the masked eye-bands (cropped photographs just showing the eyes for anonymity). These results mirrored the unmasked results. The clinic staff also narrowly preferred the hand-painted eye over the digitally-printed eye. This needs to be considered in the context of the hand-painted eyes being manufactured by a private highly-experienced ocularist, rather than the national high-throughput service (NAES). The result may have been influenced by the ocularist using high-quality colour-accurate photographs, which are not always available to the NAES. The service at LAES is being currently developed to refine the manufacturing with a focus on colour matching.

Patients and charities have helped design this study. Recruitment through charities is a novel methodology approach within this study type, which may hopefully be taken up by others. Maintaining strong patient and public involvement groups is vital for the quality and relevance of research and we aim to continue this in our future studies [13].

We identified strengths and weaknesses of the current approach to inform the design and recruitment of the future full-scale study, as well as the implementation of the service going forward. Combined with the results from the qualitative interviews, the recruitment hurdles from the patient’s perspective can be better addressed.

In this study, the participants identified the possibility of reducing the number of clinics required, refining the personnel involved in each clinic and potential patient fatigue in completing multiple patient-reported outcome measure forms as areas for improvement.

In addition to the NAES, there are around 30 local artificial eye services operating independently or as part of maxillofacial prosthetic laboratories. Technological uptake by other artificial eye services is gradual. This step-by-step approach allows the implementation of changes to long-standing services that have always used the traditional hand-painting approach.

If the novel service produces faster, better and economical artificial eyes, then full technological uptake by all service providers is the most efficient scenario. Technological uptake may often be hesitant and limited at the outset, before picking up momentum with time.

## Conclusions

Feasibility outcomes were promising in terms of eligibility, attendance and retention rates despite the inevitable impact from the Covid-19 pandemic on recruitment rates. Overall, participation in the feasibility study was well accepted by participants.

Quantitative analysis showed patient-reported outcome measures had high completion rates (91–100%) and comparable results between the two study groups. We identified the area of improvement as the resource use questionnaire data completion.

Qualitative findings suggest patients prioritise a good colour match, overall realistic appearance and timely manufacture. Further improvement is required in artificial eye appearance and fit. Importantly, we identified patient demand for improved psychological support after eye loss to help with mental health and social wellbeing. As a result, we are expanding our patient support group, which was founded by our own artificial eye users and supported by the team.

We conclude that based on the feasibility study results that a full trial is of value and achievable. Using the findings of this feasibility study, we are developing a large-scale multicentre RCT to produce definitive evidence of the effectiveness and cost-effectiveness. The findings of a large-scale RCT would benefit patients locally in the short term and NHS wide in the medium to long term.

## Summary

### What was known before

- Thousands of patients in the United Kingdom have artificial eyes, but manufacturing of artificial eyes has not changed significantly since 1948, but technological advances mean alternatives are now possible.
- Delays and colour-matching issues may severely impact a patient’s rehabilitation pathway.
- A feasibility trial was required to determine whether conducting a full-scale trial of the effectiveness and cost-effectiveness of digitally-printed artificial eyes compared to hand-painted was possible.

### What this study adds

- The feasibility study results showed that a full trial is achievable.
- A large-scale multicentre RCT is needed to produce definitive evidence of the effectiveness and cost-effectiveness.

## Supplementary information

Supplementary tables

## Data Availability

The data that support the findings of this study are available from the corresponding author, upon reasonable request.
