# Supplementary material for: A cross-over, randomised feasibility study of digitally-printed versus hand-painted artificial eyes in adults: PERSONAL-EYE-S
Source: Eye (Lond). 2024 Aug 2;38(17):3263–71. doi: 10.1038/s41433-024-03273-0 (PMC11584860; doi:10.1038/s41433-024-03273-0)
Supplement: Supplementary file 1 — Supplementary tables [file 41433_2024_3273_MOESM1_ESM.docx]

**Table A:** Follow up-rates and clinic attendance, by arm and overall

|  | Digitally-printed first (n=17) | Hand-painted first  (n=18) | Overall  (n=35) |
| --- | --- | --- | --- |
| Participants – Questionnaire completion | | | |
| Follow-up one |  |  |  |
| In follow-up, n | 17 | 17 | 34 |
| Assessment completed, n(%) | 14 (82.4%) | 16 (94.1%) | 30 (88.2%) |
| Follow-up two |  |  |  |
| In follow-up, n | 15 | 15 | 30 |
| Assessment completed, n(%) | 15 (100.0%) | 15 (100.0%) | 30 (100.0%) |
| Participants – Clinic Attendance | | | |
| Clinic one (baseline) |  |  |  |
| In follow-up, n | 17 | 18 | 35 |
| Assessment completed, n(%) | 17 (100.0%) | 18 (100.0%) | 35 (100.0%) |
| Clinic two (fitting of eye two) |  |  |  |
| In follow-up, n | 17 | 18 | 35 |
| Assessment completed, n(%) | 17 (100.0%) | 18 (100.0%) | 35 (100.0%) |
| Clinic three (Follow-up one) |  |  |  |
| In follow-up, n | 17 | 17 | 34 |
| Assessment completed, n(%) | 15 (88.2%) | 16 (94.1%) | 31 (91.2%) |
| Clinic four (fitting of eye two) |  |  |  |
| In follow-up, n | 16 | 16 | 32 |
| Assessment completed, n(%) | 15 (93.8%) | 16 (100.0%) | 31 (96.9%) |
| Clinic five (Follow-up two) |  |  |  |
| In follow-up, n | 15 | 15 | 30 |
| Assessment completed, n(%) | 15 (100.0%) | 15 (100.0%) | 30 (100.0%) |
| Close contacts – follow ups | | | |
|  | Digitally-printed first (n=3) | Hand-painted first  (n=7) | Overall  (n=10) |
| Follow-up one |  |  |  |
| In follow-up, n | 3 | 7 | 10 |
| Assessment completed, n(%) | 3 (100.0%) | 6 (85.7%) | 9 (90.0%) |
| Follow-up two |  |  |  |
| In follow-up, n | 3 | 7 | 10 |
| Assessment completed, n(%) | 3 (100.0%) | 6 (85.7%) | 9 (90.0%) |

**Table B:** raw scores at each follow-up for each outcome measure, by arm and overall

|  | Digitally-printed first (n=17) | Hand-painted first  (n=18) | Overall  (n=35) |
| --- | --- | --- | --- |
| SF-36 Physical (range 0 – 100; higher scores are better) | | | |
| Follow-up one | | | |
| N | 14 | 14 | 28 |
| Mean (SD) | 54.0 (7.1) | 52.4 (8.6) | 53.2 (7.8) |
| Median (min, max) | 56.0 (35.5, 61.5) | 54.9 (36.4, 60.9) | 55.5 (35.5, 61.5) |
| Follow-up two | | | |
| N | 15 | 13 | 28 |
| Mean (SD) | 51.4 (8.8) | 52.5 (8.0) | 51.9 (8.3) |
| Median (min, max) | 53.3 (27.5, 61.9) | 55.7 (34.9, 60.3) | 53.8 (27.5, 61.9) |
| SF-36 Mental (range 0 – 100; higher scores are better) | | | |
| Follow-up one | | | |
| N | 14 | 14 | 28 |
| Mean (SD) | 45.0 (12.5) | 48.8 (10.2) | 46.9 (11.4) |
| Median (min, max) | 42.7 (21.7, 60.8) | 52.1 (29.0, 61.5) | 49.9 (21.7, 61.5) |
| Follow-up two | | | |
| N | 15 | 13 | 28 |
| Mean (SD) | 47.9 (10.6) | 47.8 (10.2) | 47.8 (10.2) |
| Median (min, max) | 53.1 (31.2, 63.5) | 49.5 (26.7, 61.0) | 49.7 (26.7, 63.5) |
| VisQol (range 0 – 1; higher scores are worse) | | | |
| Follow-up one | | | |
| N | 14 | 15 | 27 |
| Mean (SD) | 0.85 (0.17) | 0.86 (0.16) | 0.86 (0.17) |
| Median (min, max) | 0.94 (0.54, 0.99) | 0.96 (0.53, 1.00) | 0.95 (0.53, 1.00) |
| Follow-up two | | | |
| N | 15 | 15 | 30 |
| Mean (SD) | 0.85 (0.15) | 0.84 (0.22) | 0.85 (0.19) |
| Median (min, max) | 0.87 (0.43, 0.99) | 0.91 (0.15, 1.00) | 0.90 (0.15, 1.00) |
| CD-RISC-10 (range 0 to 40; higher scores are better) | | | |
| Follow-up one | | | |
| N | 12 | 15 | 27 |
| Mean (SD) | 29.4 (9.0) | 27.2 (9.0) | 28.2 (8.9) |
| Median (min, max) | 30.5 (14.0, 40.0) | 28.0 (8.0, 39.0) | 29.0 (8.0, 40.0) |
| Follow-up two | | | |
| N | 15 | 15 | 30 |
| Mean (SD) | 28.5 (9.4) | 28.1 (8.0) | 28.3 (8.6) |
| Median (min, max) | 28.0 (12.0, 40.0) | 28.0 (13.0, 40.0) | 28.0 (12.0, 40.0) |
| DAS-24 (range 11 to 96, higher scores are worse) | | | |
| Follow-up one | | | |
| N | 14 | 16 | 30 |
| Mean (SD) | 38.9 (13.6) | 38.6 (12.9) | 38.7 (13.0) |
| Median (min, max) | 39 (21, 65) | 34.5 (19, 72) | 35.5 (19, 72) |
| Follow-up two | | | |
| N | 15 | 15 | 30 |
| Mean (SD) | 37.3 (13.9) | 38.5 (15.1) | 37.9 (14.3) |
| Median (min, max) | 36 (16, 59) | 34 (15, 74) | 35 (15, 74) |

min. = minimum, max. = maximum, SD = standard deviation.
